# Screening of *Chaetomorpha linum* Lipidic Extract as a New Potential Source of Bioactive Compounds

**DOI:** 10.3390/md17060313

**Published:** 2019-05-28

**Authors:** Loredana Stabili, Maria Immacolata Acquaviva, Federica Angilè, Rosa Anna Cavallo, Ester Cecere, Laura Del Coco, Francesco Paolo Fanizzi, Carmela Gerardi, Marcella Narracci, Antonella Petrocelli

**Affiliations:** 1 Institute of Water Research (IRSA) C.N.R, 74123 Taranto, Italy; maria.acquaviva@irsa.cnr.it (M.I.A.); rosanna.cavallo@irsa.cnr.it (R.A.C.); ester.cecere@irsa.cnr.it (E.C.); marcella.narracci@irsa.cnr.it (M.N.); 2 Department of Science and Biological and Environmental Technologies, University of Salento, 72100 Lecce, Italy; federica.angile@unisalento.it (F.A.); laura.delcoco@unisalento.it (L.D.C.); fp.fanizzi@unisalento.it (F.P.F.); 3 Institute of Sciences of Food Production, U.O.S. di Lecce, Via Prov.le Lecce-Monteroni, 72100 Lecce, Italy; carmela.gerardi@ispa.cnr.it

**Keywords:** antibacterial activity, antioxidant activity, NMR spectroscopy, lipidic extract, macroalgae

## Abstract

Recent studies have shown that marine algae represent a great source of natural compounds with several properties. The lipidic extract of the seaweed *Chaetomorpha linum* (Chlorophyta, Cladophorales), one of the dominant species in the Mar Piccolo of Taranto (Mediterranean, Ionian Sea), revealed an antibacterial activity against *Vibrio ordalii* and *Vibrio vulnificus*, common pathogens in aquaculture, suggesting its potential employment to control fish and shellfish diseases due to vibriosis and to reduce the public health hazards related to antibiotic use in aquaculture. This extract showed also an antioxidant activity, corresponding to 170.960 ± 16. mmol Trolox equivalent/g (oxygen radical absorbance capacity assay—ORAC) and to 30.554 ± 2.30 mmol Trolox equivalent/g (Trolox equivalent antioxidant capacity assay—TEAC). The chemical characterization of the extract, performed by 1D and 2D NMR spectroscopy, highlighted the presence of free, saturated (SAFAs), unsaturated (UFAs) and polyunsaturated (PUFAs) fatty acids. The high content of ω-6 and ω-3 PUFAs confirmed also by gas chromatography indicates the potentiality of this algal species in the production of fortified food. The antibacterial activity seems related to the presence of linolenic acid present at high density, while the antioxidant activity could be likely ascribable to molecules such as carotenoids and chlorophylls (characterized also by thin-layer chromatography), known for this property. The presence of polyhydroxybutyrate, a biopolymer with potentiality in the field of biodegradable bioplastics was also detected. The exploitation of *C. linum* for a future biotechnological application is also encouraged by the results from a first attempt of cultivating this species in an integrated multi-trophic aquaculture (IMTA) system.

## 1. Introduction

About 70% of our planet is covered by oceans [1], hosting an impressive wealth of biodiversity, which offers crucial ecosystem services. This astonishing biodiversity associated with as much a high chemical diversity represents a repository of new bioactive molecules, which exhibit peculiarities different from terrestrial natural products and are potentially suitable for use in the industry of drugs, cosmetics, nutritional supplements and molecular probes [2,3,4,5]. In the last years, more than a thousand of pharmacologically active compounds of marine origin have been isolated and characterized from different organisms [6,7]. As a further application, several compounds from seaweeds proved to be also a useful ecofriendly tool in the control of both the environmental quality and the oxidative stress [8,9]. Generally, algal secondary metabolites are mostly involved in the chemical defense against several biotic enemies including grazers and epibionts [10] even though they are also synthesized in response to ecological pressures in the surrounding environment (e.g., desiccation, nutrient availability and UV) [11]. In some cases the effect of these metabolites can be direct, such as the impaired survival of sea urchins living and feeding on seaweeds in Australia [12]. In other cases, seaweeds can make themselves disgusting to herbivores, combining forces with deterrent organisms [13]. Macroalgae contain diverse groups of bioactive chemicals such as macrolides, polysaccharides, minerals, vitamins, proteins, lipids, polyphenols and fatty acids with antibacterial, antiviral and antifungal properties [7,14]. These potential drugs, especially antibiotics, are now attracting considerable attention from the pharmaceutical industries due to the need to contrast the antibiotic resistance of pathogens [15,16]. Indeed, the indiscriminate and prolonged employment of antimicrobial drugs has produced therapeutic failures coupled with the selection of resistant pathogens [17,18]. Although considerable advancement is being performed within the fields of chemical and engineered biosynthesis of antimicrobials, nature still represents the richest and the most versatile source for new antibiotics [19,20,21]. Even though the therapeutic value of marine algae was recognized since millennia in traditional medicine [22], only recently modern screening methods have identified antibacterial compounds in the secondary metabolites of algae. Several studies have shown molecules from green, brown and red marine algae possessing in vitro capabilities of inhibiting bacteria, viruses, fungi and other epibionts [4,23,24,25,26]. Much of these researches concerned the inhibition of human pathogens by algal extracts, while reports on the effects against fish pathogenic bacteria are less numerous and also more recent [27,28].

Marine algae represent also a great source of antioxidants [29]. Compounds with antioxidant activity have been found in brown, red and green algae. Concern over the safety of synthetic antioxidants has also led to increased interest on natural compounds of this kind. Indeed, the use of synthetic antioxidants such as butylated hydroxytoluene (BHT), butylated hydroxyanisole (BHA), propyl gallate (PG) and tert-butylthydroquinone (TBHO) has been restricted because of their potential toxic effect on humans and have led to increased interest on natural antioxidants, because of their safety properties and wide distribution [30]. 

The genus *Chaetomorpha* (Chlorophyta, Cladophorales), nomen omen “stiff hairs” [31], is characterized by unbranched heavy filaments [32]. It includes 70 species [33], mostly containing bioactive compounds, which make them usable in various applications. Some species were analyzed to detect the chemical nature of those compounds, so that some of them resulted in being edible, due to their content in nourishing substances [34], some others showed antioxidant activity [35] or a noticeable content in fatty acids [36,37]. In the Mediterranean, six species of the genus *Chaetomorpha* are present [38]. Among them, *Chaetomorpha linum* (O.F. Müller) Kützing is the most widespread and studied from an ecological perspective [39,40], but recently also for the biotechnological applications, such as the employment of its extracts in animal disease control [41] or in cosmetic industry [31].

In the Mar Piccolo of Taranto (southern Italy, Mediterranean Sea), *Chaetomorpha linum* is one of the dominant species, which can reach considerable standing crops yearly [42]. In the light of possible applications in medicine, dietary supplements, food industries or cosmetics, the lipidic extract of *C. linum* was chemically characterized through 1D and 2D multidimensional NMR spectroscopy, gas chromatography (GC) and thin-layer chromatography (TLC). Since the industry requires a considerable amount of biomass for the extraction of secondary metabolites, the seaweed cultivation trials to obtain *C. linum* biomass in an integrated multi-trophic aquaculture (IMTA) system are described.

## 2. Results

### 2.1. Antimicrobial Activity

**Figure 1:**
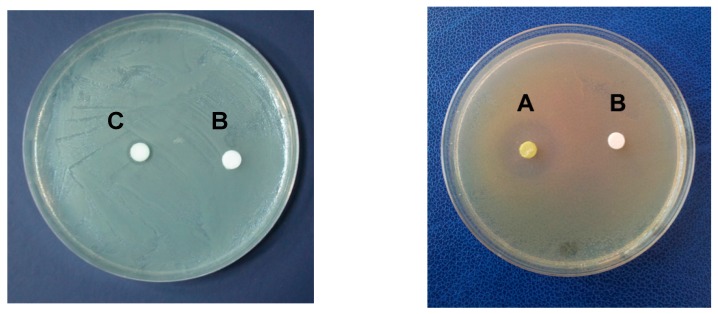
Disc diffusion assay. *Chaetomorpha linum* lipidic extract against *Vibrio ordalii*. (**A**) Disc impregnated with 100 μL algal extract; (**C**) disc impregnated with 5 μL algal extract and the (**B**) negative control.

The in vitro assays highlighted the presence of antimicrobial activity in *C. linum* lipidic extract, which proved effective against *Vibrio ordalii* and *V. vulnificus*. Conversely, it was ineffective against *V. alginolyticus*, *V. harveyi*, *V. mediterranei*, *V. parahaemolyticus* and *V. salmonicida*, as well as against all the tested yeasts and the human pathogens *Enterococcus* sp., *Pseudomonas* sp., *Staphylococcus* sp. and *Streptococcus agalactiae* (Table 1, the diameters of inhibition zones were used as a measure of the degree of the antimicrobial activity on each strain). In particular, tests performed against *V. ordalii* and *V. vulnificus* were able to demonstrate antibacterial activity. The diameter of the growth inhibition was 8 mm employing 5 μL of the algal extract (corresponding to 25 μg of dry extract) and reached 12 mm with 10 μL of the algal extract (corresponding to 50 μg of dry extract, Figure 1). Moreover, pure α-linolenic acid (≥99, Sigma-Aldrich) was able to inhibit the growth of *V. ordalii* and *V. vulnificus* by the in vitro assay starting from 0.018 mg mL^−1^ corresponding to the minimal inhibitory concentration (MIC). 

### 2.2. Antioxidant Activity

Trolox equivalent antioxidant capacity (TEAC) and oxygen radical absorbance capacity (ORAC) assays were performed to test the antioxidant activity of the lipid extract of *C. linum*. By the former assay the measured antioxidant capacity resulted in being six times lower than the one measured by the latter assay. Moreover, to complete the evaluation of the antioxidant capacity, the Folin–Ciocalteu (F–C) assay was performed. The results are reported in Table 2.

### 2.3. Fatty Acid Profile

**Figure 2:**
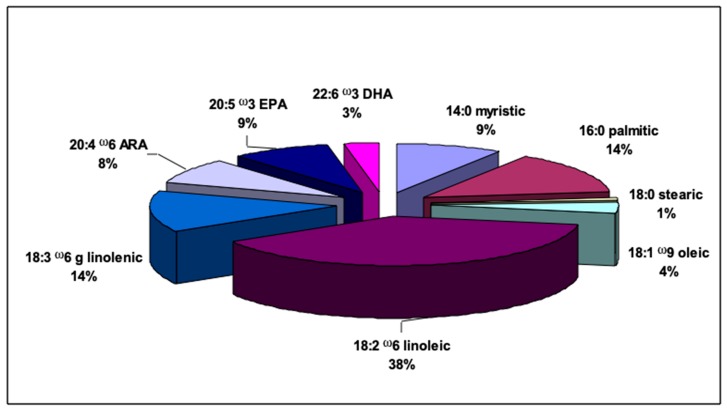
Fatty acid profile (% of total fatty acids) of *Chaetomorpha linum* collected in the Mar Piccolo of Taranto.

The fatty acid profile of total lipids extracted from *C. linum* is reported in Figure 2. Among the total fatty acids (FAs), polyunsaturated fatty acids (PUFAs) resulted in the most abundant reaching a value of 71.97%. Linoleic acid (18:2 ω-6), eicosapentaenoic acid (EPA, 20:5 ω-3) and arachidonic acid (AA, 20:4 ω-6) were the most abundant PUFAs accounting for 38.46%, 8.83% and 8.14% of total FAs, respectively. The ω-3 fatty acid docosahexaenoic (DHA, 22:6 ω-3) represented 2.91%. Saturated fatty acids (SAFAs) represented 23.83% of the total fatty acids (FAs). Palmitic acid methyl ester (16:0) was the prevalent SAFA (14.03% of total FAs), followed by the myristic acid methyl ester (9% of total FAs). Monounsaturated fatty acids (MUFAs) showed the lowest percentage (4.2% of total FAs) and among them oleic acid methyl ester (18:1 ω-9) prevailed. The ratio of ω-3 to ω-6 fatty acids was <1.

### 2.4. NMR Spectroscopy

**Figure 3:**
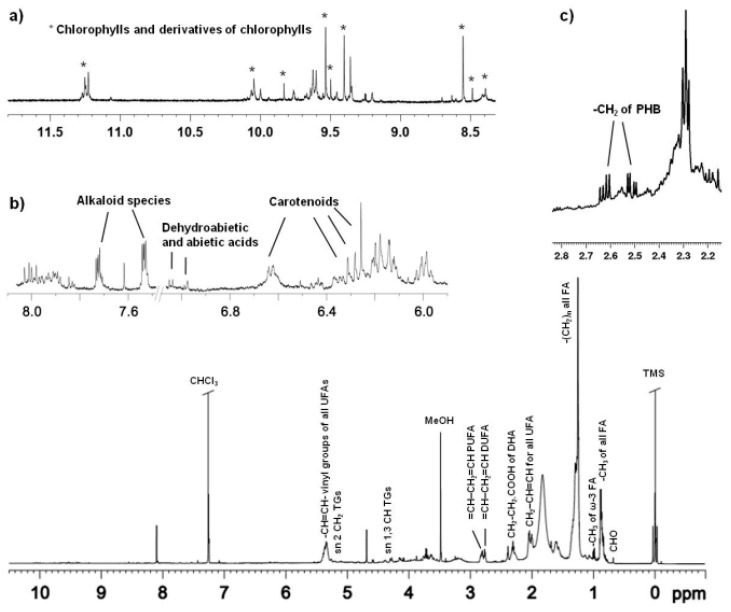
Typical ^1^H NMR spectrum of *C. linum* lipid extract, with expansions for (**a**) tetraphyrrolic region of chlorophylls and derivatives (*); (**b**) conjugated double bond region of carotenoids and (**c**) PHB region.

A typical one dimensional (1D) ^1^H NMR spectrum of lipid extract of *C. linum* is shown in Figure 3. The assignments reported in the ^1^H NMR spectrum and Table 3 were obtained on the basis of analysis of the 2D NMR spectra (2D ^1^H JRES, ^1^H ^1^H COSY, ^1^H ^13^C HSQC and HMBC) and by comparison with published data [3,4,26,31,43,44,45,46,47,48,49,50].

The ^1^H NMR spectrum of the algae lipid extract shows the characteristic signals of sterols, triacylglycerols (TGs), saturated and unsaturated fatty acids (SAFAs and UFAs, respectively). The presence of cholesterol (CHO) was indicated by signals at low frequencies, in particular 0.68 ppm (^13^C 11.67 ppm), 0.86 (^13^C 22.45–22.38 ppm), 0.92 ppm (^13^C 18.56) and 1.01 ppm, (^13^C 19.06 ppm) [43]. Signals at 4.28, 4.15 (^13^C 62.01 ppm) and 5.26 ppm (^13^C 68.6 ppm) were ascribed to CH sn-1,3 and CH_2_ sn-2 of glycerol moiety of triacylglycerols (TGs), while the presence of monoacyl (MAGs) and sn-1,2/2,3diacilglycerols (DAGs) was confirmed by the signals at 3.63 ppm (^13^C 70.5 ppm) for MAGs and multiplets at 3.73 (HO–CH_2_–CH–) and 5.08 ppm (2′-CHOCO–), for DAGs [4,44,45]. Moreover, the signals in the range 0.97–1.02 ppm (^13^C 14.18 ppm) were assigned to the terminal methyl groups of all FA, in particular SAFA and UFA, such as oleic (MUFA ω-9), linoleic (DUFA ω-6), linolenic and among PUFA ω-3, docosahexaenoic acid. The multiplets at 1.32–1.22 ppm and 1.68–1.46 ppm were assigned to methylene protons of all alkyl chains (–(CH_2_)_n_–) and CH_2_ in β position with respect to the carboxylic acid esters of all FA (COOCH_2_CH_2_). The vinyl protons, CH=CH, of all UFAs were found in the range 5.42–5.30 ppm. The allylic protons (CH=CH–CH_2_), in α position respective to vinyl groups of all UFAs, resonated at 2.07–1.98 ppm (with resonances at 2.01 ppm for MUFA ω-9, 2.03 for PUFA ω-6 and 2.07 for PUFA ω-3, ^13^C 27.2 ppm) and methylene groups in α to C=O of all FA were found in the range 2.38–2.32 ppm. Moreover, signals in the range 2.42–2.38 ppm (with ^13^C resonances at 22.59 ppm and 34.07 ppm), corresponded to CH_2_–CH_2_–COOH of docosahexaenoic acid (DHA), partially overlapped with CH_2_–COOH of arachidonic acid (ARA). The presence of PUFA ω-3 (DHA and linolenic acids) and DUFA (linoleic acid) was also confirmed by signals of methylenic protons CH_2_ at 2.86–2.78 and 2.78–2.73 ppm (^13^C 25.6 ppm). PUFAs (mainly DHA, ARA, linolenic and linoleic acids) percentages were calculated by the integration of the corresponding selected NMR signals. In particular, peaks at 0.98 ppm (CH_3_ terminal of ω-3 fatty acids), 2.38–2.32 ppm (methylene groups in α to C=O of all FA), 2.42–2.38 ppm (CH_2_–CH_2_–COOH of DHA and CH_2_–COOH of ARA), 2.86–2.78 ppm and 2.78–2.73 ppm (CH_2_ of PUFA ω-3 and DUFA) were integrated with respect to the internal standard (TMS) [44]. A content values of 10% for the sum of DHA and ARA, 13.63% for linolenic and 38.87% for linoleic acids were obtained. The bis-allylic protons of linolenic acid appeared more deshielded than in linoleic acid, due to a larger number of double bonds in the acyl chain [44]. In addition, by 2D ^1^H ^1^H COSY, HSQC, HMBC spectra (Figure 3) analysis, the characteristic signals of poly-β-hydroxybutyrate (PHB) were identified, in particular two doublet at 2.58 ppm and 2.48 ppm (^13^C HSQC 40.75 ppm and HMBC ^13^C 169.9 ppm), attributed to the methylene group, coupled with the methyl group at 1.26 ppm and the methine at 5.23 ppm as already reported in Stabili et al. [26]. Moreover, a set of aromatic signals was detected, consistent with the presence of dehydroabietic and abietic acids (7.16 ppm, 7.00 ppm and 6.88 ppm for ^1^H, and 125.57 ppm and 127.28 ppm for ^13^C resonances) [31,46,47]. The signals at 7.53 ppm (^13^C 130.88 ppm) and 7.72 ppm (^13^C 128.90 ppm) were assigned to aromatic protons of alkaloid species [3]. The signals in the downfield frequencies between 11.5 ppm and 8.5 ppm corresponded to tetraphyrrolic region of chlorophylls (Figure 3a), while their signals in the upfield part of the ^1^H NMR spectrum (typically found at −1.43 ppm and −1.61 ppm) were absent in the CDCl_3_/CD_3_OD mixture because of an exchange with the hydroxyl group of methanol [48]. In particular, signals at 11.25 ppm, 11.23 ppm, 10.04 ppm, 9.99 ppm and 9.83 ppm were assigned to chlorophyll b, while intense signals at 9.54 ppm and 8.55 ppm were assigned to chlorophyll a. Other signals at 9.40 ppm and 9.35 ppm and 9.62 ppm and 9.60 ppm have been assigned to chlorophyll derivatives, such as pheophytin a and b [48,49]. A complex pattern of signals in the range 6.70–6.00 ppm were assigned to conjugated double bonds of carotenoids, such as carotenes and xanthophylls [50,51]. In particular, β-carotene and lutein have been identified by peaks in the range of 6.69–6.57 ppm and 6.32–6.13 ppm [48,49], with overlapping signals of chlorophylls and chlorophylls derivatives [49] (as shown in Figure 3b). Interestingly, pigments NMR identification and quantification offers several advantages with respect to other analytical techniques. According to literature, although the pigment analysis by some HPLC methods is fast, these methods can be complex, labor intensive and time-consuming. Moreover, many labile substances could undergo decomposition or modification during the chromatographic separation step [49].

### 2.5. Thin-Layer Chromatography

**Figure 4:**
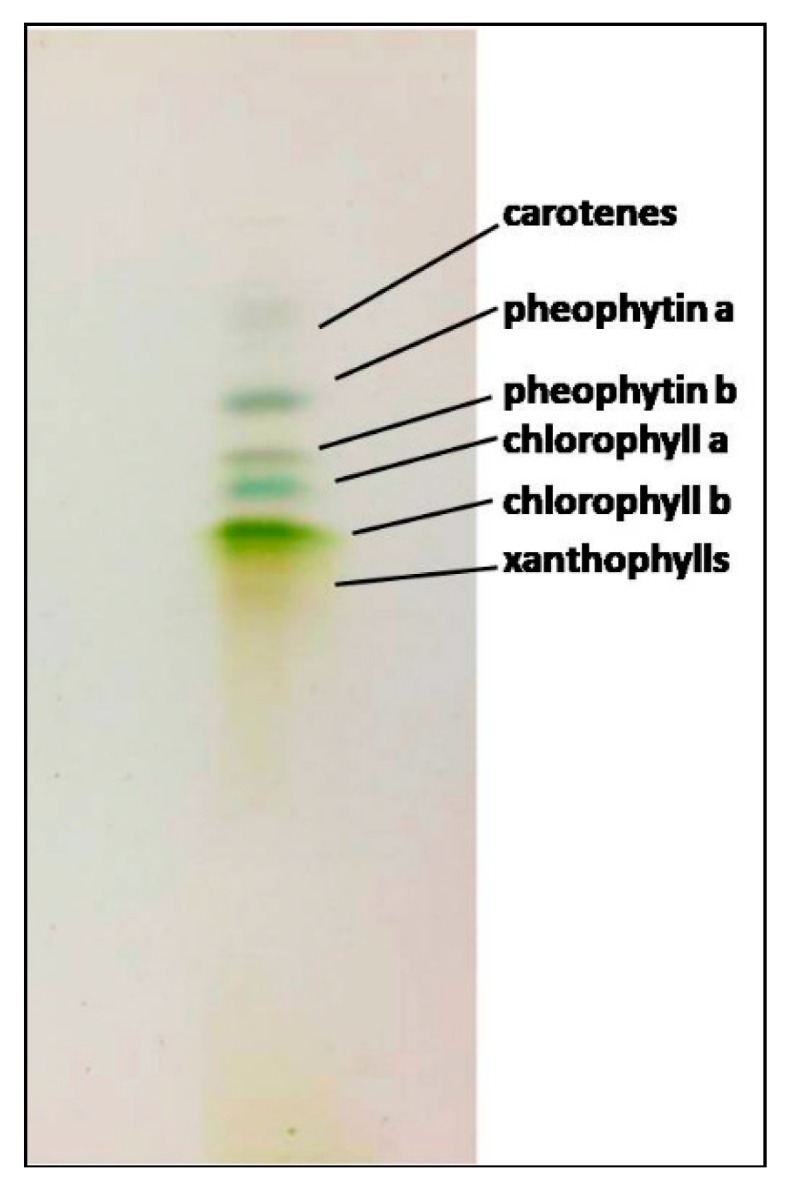
Thin-layer chromatography (TLC) plate of the *C. linum* lipidic extract. Carotenes, yellow-orange band; pheophytin a and b, gray bands; chlorophyll a, blue-green band; chlorophyll b, green band and xanthophylls, yellow bands.

Thin-layer chromatography (TLC) analysis was performed on *C. linum* lipidic extract (Figure 4), confirming the presence of the pigments evidenced by NMR identification. In particular, four distinct bands, corresponding to different chlorophylls, were identified: Blue-green, green and gray bands were recognized for chlorophylls (a and b) and pheophytins (a and b), while two blurry bands (extremely up, yellow-orange and down, extended yellow bands) corresponded to carotenoids The total content of chlorophylls and carotenoids was also calculated, by the integration of unbiased signals in the ^1^H NMR spectrum, obtaining 2.7 × 10^−4^ mg/g and 6.30 × 10^−5^ mg/g, respectively.

### 2.6. Chaetomorpha Linum Cultivation Trials

**Figure 5:**
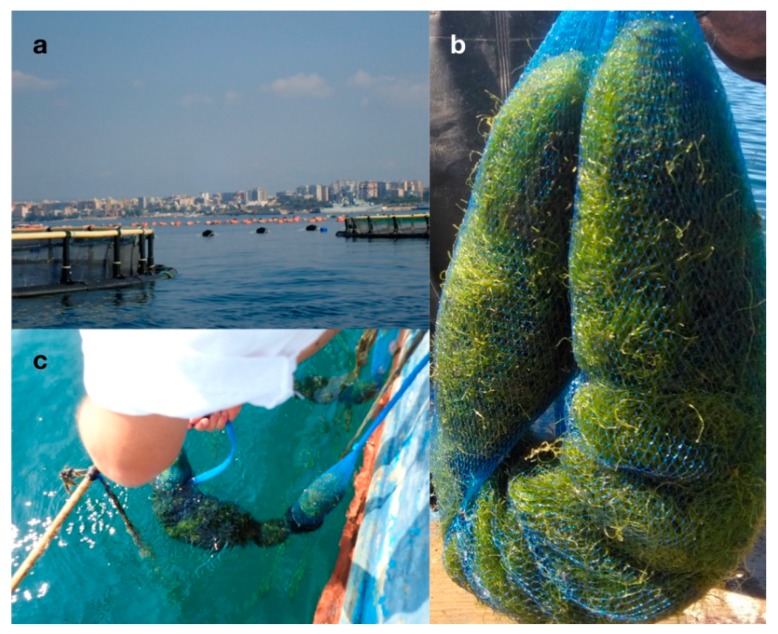
Cultivation trials of *Chaetomorpha linum* in the Mar Grande of Taranto. (**a**) The integrated multi-trophic aquaculture (IMTA) system; (**b**) *Chaetomorpha linum* located in nets and (**c**) suspended nets.

The cultivation of *C. linum* in the aquaculture plant (Figure 5) gave a biomass increase of about 5% specific growth rate (SGR) every month.

## 3. Discussion

Seaweeds contain a wide range of bioactive compounds, several of which find commercial applications in the pharmaceutical, medical, cosmetic, food industry and in agriculture fields [9]. In this connection, studies on the seaweeds from the Mar Piccolo of Taranto have already been in progress for several years [3,4]. In *C. linum*, the detection, for the first time, of an antibacterial activity against *Vibrio ordalii* and *V. vulnificus* was a noteworthy result. Indeed, this evidenced antibacterial activity against vibrios is of high interest since the animal, mainly fish and shellfish [52], and human diseases called vibriosis can be due to several *Vibrio* species [53]. This result is crucial to a greater extent taking into account that:

1. *Vibrio ordalii*, previously designated as *V. anguillarum* biotype 2, causes worldwide serious haemorrhagic septicaemia inducing mortality in fish including cultured Atlantic salmon, Pacific salmon, rainbow trout, rockfish and gilthead sea bream [54,55,56,57]. *Vibrio vulnificus* is an opportunistic human pathogen causing three distinct syndromes: Primary septicemia, wound infection and gastroenteritis in susceptible individuals [58]. This species is highly lethal and is also responsible for several seafood-related deaths [59]. Similarly to other seafood-borne bacteria, it accumulates in the tissues of filter-feeders invertebrates such as oysters, clams and mussels. Infections are often acquired from eating raw oysters [60];
2. Vibriosis are the cause of considerable economic losses in aquaculture activities worldwide, close to nine billion US dollars per year according to the most recent appraisals [61,62,63];
3. The production of fish in aquaculture plants is becoming more and more necessary due to the increasing demand of fish food for a growing world population, especially in developing countries [63];
4. The increase in production caused an increase in the use of antibiotics to contend with fish diseases. The main outcome of this growth was surely the development of an antibiotic resistance in the same fish and also in human consumers. Moreover, since antibiotics are served to fish through medicated feed, the excessive release in the environment of feed residues caused accumulation of antibiotics also into the sediments and the resultant selection of resistant microorganisms [64].

Thus, the research of unconventional substances, mainly eco-friendly feed additives, to combat fish diseases is ongoing [65]. Seaweeds seem a good alternative therapeutic source [66], and *C. linum* seems a good candidate for exploitation in this field.

Neither yeasts (i.e., *C. albicans* and *C. glabrata*) nor the human pathogens (i.e., *Enterococcus* sp., *E. coli*, *Staphylococcus* sp. and *Streptococcus* sp.) tested with the lipid extract of *C. linum* showed sensitivity. However, it must be emphasized that in the present study we analyzed the antibacterial activity of the lipidic extract, but further research will be undertaken in order to evaluate whether the crude extract or the aqueous extract of this algal species could prove effective on other microorganisms or capable of other relevant biological activities.

Another interesting result transpired from the analysis of the lipid extract of *C. linum* was the presence of an in vitro antioxidant activity. TEAC, ORAC and F–C assays were jointly used to assess this antioxidant capacity because the use of a single method for the detection of antioxidants could underestimate or even overlook their content [67,68].

In order to understand the possible biotechnological applications, and then which kind of compounds could be responsible for the detected activities, the chemical characterization of the extract was carried out. Therefore, gas chromatography, thin-layer chromatography and NMR spectroscopy were performed aiming to highlight the possible nutritional value as well as the antibacterial and antioxidant activity of *C. linum* lipid extract. Multinuclear and multidimensional NMR spectroscopy was employed in the present study since, among the available analytical tools for this purpose, it recently proved to be more sensitive, as well as easier and quicker [51]. Data on fatty acids from gas chromatographic analysis were compared with those obtained by NMR analysis and both displayed the presence of SAFAs and UFAs among fatty acids with similar percentages. In addition NMR analysis revealed also the presence of glycerol moieties of monoacyl (MAGs), diacylglycerols (DAGs) and triacylglycerols (TGs). According to the existing literature [69,70], the content of SAFAs in *C. linum* resulted higher than UFAs. The chemical composition of extracts from *C. linum* growing wild in Corsican pond, were already investigated by GC–MS (derivatization) and ^13^C NMR spectroscopy [31]. In that study eighteen compounds were identified from both pentane and ethyl acetate extract and in particular, accordingly with our results, fatty acids, mainly saturated, resulted as the main compounds from the pentane extract. ^13^C NMR analysis was also useful to detect components of the sterols family, including cholesterol, as the major compounds from the ethyl acetate extract. In the present study, based on ^1^H NMR analysis, the presence of cholesterol was also recorded. Among fatty acids, oleic, linoleic, linolenic and docosahexaenoic (DHA) acids prevailed in the lipidic extract of *C. linum* grown in the Mar Piccolo. Significant levels of oleic acid were already observed in the red seaweeds *Gracilariopsis longissima*, *Gracilaria incurvata* [71], *Gracilaria tikvahiae*, *Gracilaria corticata* [72], *G. verrucosa* [73] and *Iridaea cordata* [74]. Moreover, Van Ginneken et al. [75] analyzed the fatty acid composition of nine seaweeds (four brown, three red and two green) and found DHA in the brown *Sargassum natans*. Some of the essential polyunsaturated fatty acids (PUFAs), such as arachidonic acid and DHA, are components of the brain membrane phospholipids. Mammals are unable to produce them and consequently they must be supplied as a food supplement, considering that inappropriate fatty acids consumption is the major cause of human chronic diseases. Fish oil and animal food sources contain PUFAs ω-3, by contrast vegetable oils mainly furnish PUFAs ω-6. In particular, DHA showed to have beneficial effects on preventing human cardiovascular diseases, cancer, schizophrenia, and Alzheimer’s disease [76]. Moreover, this ω-3 fatty acid is necessary in the growth and functional development of the brain as well as in the maintenance of the normal brain function in adults. In this framework, it must be underlined that commercial production of DHA, and also of eicosapentaenoic acid (EPA) from algae became viable in the last part of the 20th century due to the increase in awareness of their benefits for health. Indeed, marine macroalgae proved to be an excellent wellspring of PUFAs with a ω6 FA: ω3 FA ratio less than 10, which the World Health Organization (WHO) strongly recommends to be achieved by the ingestion of some edible sources rich in ω 3 and ω6, useful to avoid inflammatory, cardiovascular and neuro-chronic sickness [77]. Thus, on account of our results we suggest that *C. linum* could be used as a natural source of fatty acids extracted from algae to be used in fortified foods. Moreover, the biomass could be used directly as a feed additive in various animal industries such as poultry farms or fish farms, as already tested for other algae [78,79]. Indeed, due to the rising cost of fish feeds worldwide, every innovative natural resource must be taken into account as a potential ingredient in their preparation, and algae are considered suitable alternative sources of protein and lipid for farmed fish because it was observed that the addition of small amounts of several algal-based meal to fish diets has produced positive effects on growth, feed utilization, lipid metabolism, liver function, body composition, stress responses and disease resistance [80]. 

Another important application of fatty acids from seaweeds could be linked to their use as antibacterial agents [3,4,8]. In algae, oleic, linoleic and linolenic acids are the major component of UFAs, which show antibacterial activity and the capability to inhibit pathogenic bacteria growth [4,69,70]. These properties seem to be attributable to the capacity of the above mentioned long chain PUFAs to interfere in the synthesis of bacterial FAs, and are related to incubation time, concentration and FAs unsaturation degree [70]. The antibacterial activity against *Vibrio ordalii* and *V. vulnificus* in *C. linum* from the Mar Piccolo seems related to the occurrence of linolenic acid. Indeed, the growth of *V. ordalii* and *V. vulnificus*, by the in vitro assay, resulted in being inhibited by pure α-linolenic acid. This result is consistent with the high percentage of this fatty acid (about 38%) recovered by NMR and GC analyses. 

In addition to fatty acids, several other secondary metabolites from seaweeds showed antibacterial activity, such as alkaloids, halogenated compounds, lectins, phlorotannins, pigments and polysaccharides. In this framework, it must be also underlined that the NMR analysis of the *C. linum* extract showed the presence of terpenes moieties, that are mainly identified in dietary and herbal plants and are important defense compounds [81]. For example, the dehydrodiabetic acid is a diterpene resin acid and presents several biological actions such as antimicrobial, antitumor, antiviral and cytotoxic activities [81], while the abietic acid shows bacteriolytic action associated with interaction and lysis of cell membranes [47].

Concerning the antioxidant activity, since the ORAC assay showed a higher activity than that resulted from the TEAC assay, and it is known that the antioxidant capacity of carotenoids is higher when tested by ORAC than by a TEAC assay [82], it is presumable that in *C. linum* it could be related to the carotenoid molecules, which were identified by NMR analysis, together with a phenolic content similar to those detected in some brown algae, already known for their high antioxidant potential [83]. Furthermore, Cerón et al. [84] demonstrated that the fatty acid profile contributes to the antioxidant capacity of algal lipidic extract. In particular, carotenoids esterified with oleic acid show higher antioxidant capacity than free carotenoids. Those compounds were here identified by NMR and CG analysis in the *C. linum* lipidic extract, and thus they could be involved in the evidenced activity. Moreover, a contribution of chlorophylls and above all of their derivatives pheophytin a and pheophytin b, identified by NMR and TLC analysis in the lipidic extract of *C. linum*, can be invoked on the in vitro tested antioxidant capacity as already demonstrated in the case of plant extract, virgin olive oils, green tea and some algae [85,86]. Biological activities attributed to chlorophyll derivatives are consistent with prevention of oxidative DNA damage and lipid peroxidation. The antioxidant activity and the contents of total phenolics and flavonoids were already quantified in the methanolic extracts of *C. linum* collected along the northern coasts of the Persian Gulf in southern Iran [35]. Phenolic content of the extract analyzed in this study was much higher than the value recorded for the Persian Gulf, while antioxidant activity was not comparable because different assays were used. Compounds with antioxidant activity have been found in several species of brown, red and green algae [29]. Since concern about the safety of the synthetic antioxidants, on a pair with antibiotics, have led to increased interest on natural antioxidants, which are commonly found in plants and seaweeds, these compounds could be used in the formulation of drugs useful in the treatment of a number of diseases. Indeed, as already demonstrated in the study of Lanfer-Marquez et al. [87], oxidative stress plays an important role in the pathogenesis of atherosclerosis, alcoholic liver cirrhosis, cancer, etc. and it is started by free radicals, especially reactive oxygen species (ROS).

Last but not least, also the presence of polyhydroxybutyrate (PHB) resulted from the NMR analysis of the ^1^H NMR spectrum in CDCl3, already detected in other macroalgae [3,4,26], but for the first time in *C. linum*. As it is well known, PHB is a linear polymer commonly synthesized by bacteria in the form of reserve granules [88], which for its biodegradability, in other words the capacity of decomposing into simplest molecules such as CO_2_, CH_4_, H_2_O, and residual biomass is considered a biocompatible substance. This result is particularly intriguing considering that the seaweed cultivation trials to obtain *C. linum* biomass in the IMTA system realized in the northern Ionian Sea is furnishing promising yields of seaweed from which this bio polymer could be extracted and, above all, that PHB could be used in the production of compostable bioplastics, considered the only alternative to the conventional petroleum based plastic. Indeed, it is now obvious that this last represents a serious environmental problem due to the release of hydrocarbons into the atmosphere and the resistance to natural or biological decomposition with consequent ocean plastic pollution [89]. In addition, the perspective area of PHB application includes the production of several medical devices in the field of biodegradable screws and plates for cartilage and bone, membranes useful in periodontal treatment, surgical dental sutures, orthopaedic, hernioplasty and skin surgery [87,90]. It is well known that corn, wheat, sugar beets and sugar cane are employed to develop next-generation bioplastics. Even though these plastics are environmentally friendlier compared to those that are fossil fuel-based of current use, in their production there is competition for land between crop used for bioplastic and those used for food [91]. Conversely, seaweeds can be directly cultivated in the marine environment, and if grown in sustainable aquaculture plants, as the case of *C. linum*, could also bioremediate water and possibly achieve high productivity, giving biomass useful for different purposes, among which also the production of seaweed bioplastic, which could represent a good chance in the fight against plastic pollution [92]. This aim will be achieved when important progress in the bioplastics industries will be accomplished through the development of a specific technology. 

In conclusion, the results obtained in the present study can offer an optimistic expectation in the possible exploitation of *C. linum* for biotechnological applications, due to its contents in a high variety of useful secondary metabolites. The employed metabolomic approach based on nuclear magnetic resonance spectroscopy allowed one shot multicomponent detection in complex mixture represented by the *C. linum* lipidic extract. In this framework further studies will be undertaken to deeply investigate the *C. linum* lipidic extract providing not only the detection but also the possible isolation of interesting molecules performed by the combination of different analytical techniques such as HPLC, GC–MS and LC–MS methods. The chemical isolation and purification of the effectors involved in the antibacterial and antioxidant activity will be particularly useful to further elucidate their mode of action. Obviously, no definite conclusion can be achieved at this stage about the possible transfer at an industrial level because several preliminary evaluations are necessary, such as the assessment of the possible toxicity of the compounds, the process for drying algae and making pellets, and especially the possibility of making high algal biomass amounts available. To overcome this last aspect, cultivation of *C*. *linum* might represent a solution. Seaweed cultivation is a growing worldwide industry, and when performed in IMTA systems with edible and non edible marine organisms (e.g., fish, mussels, sponges and polychaetes), as in our case, can represent also a bioremediation tool to reduce the environmental impact deriving from aquaculture activities, thus constituting an added value. Our first attempts of cultivating *C. linum* in IMTA have given encouraging results with a medium monthly increase of about 5% and abatement of the nitrogen salts in the culture medium that lead to hope for a future biotechnological application of the investigated macroalga.

## 4. Materials and Methods

### 4.1. Species Description and Collection

**Figure 6:**
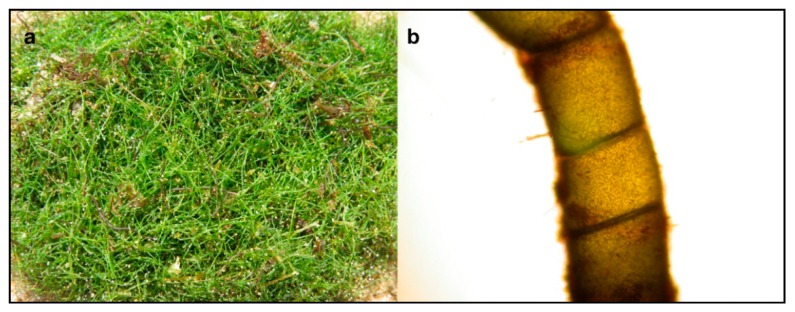
*Chaetomorpha linum* from the Mar Piccolo of Taranto. (**a**) Thallus on the bottom and (**b**) particular of a cell showing the thin cell wall.

*Chaetomorpha linum* has a bright green unbranched filamentous thallus made up of cylindrical cells up to two to three times longer than larger, with a thin cell wall (Figure 6a,b). Filaments can reach several meters in length and are generally unattached, making thick entangled cordons lying on the bottom.

**Figure 7:**
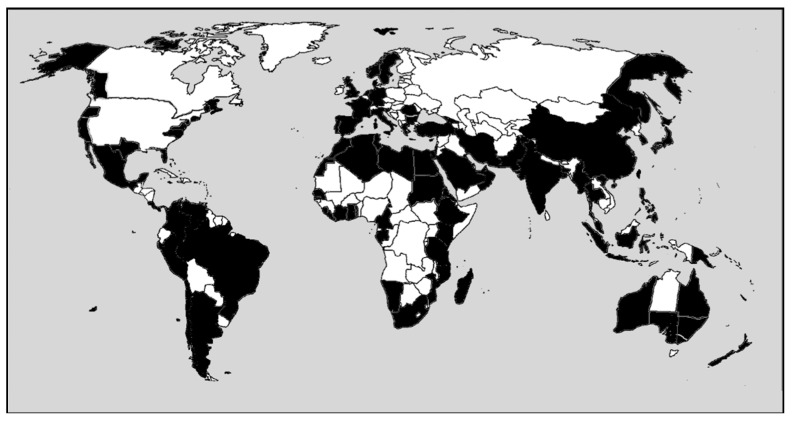
World Distribution of *Chaetomorpha linum*. In black countries where the species is reported.

This species has a worldwide distribution, from tropical to arctic zones (Figure 7), mainly in shallow brackish waters.

In the Mar Piccolo of Taranto, *Chaetomorpha linum* shows a seasonal cycle with a period of vegetative growth between late winter to early spring alternated with a period of vegetative rest (a quasi-complete withdrawal) in late summer. 

Thalli of *C. linum* were collected in the basin, at 50 cm of depth during the season of maximum growth. Three replicates of about 500 g of fresh material were handpicked and transferred into aseptic containers to the laboratory under controlled temperature (4 °C). In the laboratory the species was easily identified through the examination of the diacritic morphological characters.

### 4.2. Preparation of Lipidic Extracts from the Macroalga

By using a mixture of ethanol and (40%) and sodium hypochlorite (1%) for 10 s the algae samples were cleaned, in order to eliminate epiphytes and other marine organisms [93]. After removal of the necrotic parts, the same samples were rinsed with sterile sea water to eliminate any other associated debris. The cleaned material, after air-dried was pulverized. Extraction was carried out, with a soxhlet apparatus, using 3 g of each sample and 150 mL of chloroform/methanol (2:1 at 55–60 °C for 24 h). Extraction solvents were removed under vacuum at controlled and constant temperature, then absolute ethanol (95%, from J.T. Baker, from Avantor, Radnor, PA, USA) was added to obtain a final concentration of 5 mg/mL of extract in ethanol. Finally, the antimicrobial and antioxidant activities were assayed.

### 4.3. Test Microorganisms

Seven human pathogenic microbial strains were used to test antibacterial activity (*Candida albicans*, *Candida famata*, *Candida glabrata*, *Enterococcus* sp., *Pseudomonas* sp., *Staphylococcus* sp. and *Streptococcus agalactiae)* in addition to several *Vibrio* strains isolated and identified from seawater samples of the Mar Piccolo of Taranto (*Vibrio alginolyticus*, *Vibrio harveyi*, *Vibrio mediterranei*, *Vibrio ordalii*, *Vibrio parahaemolyticus*, *Vibrio salmonicida* and *Vibrio vulnificus*) [94,95].

### 4.4. Antimicrobial Activity

Kirby Bauer method [96] was used to evaluate the antimicrobial activity. Sterile 6 mm diameter paper discs (AA, Whatman International Ltd., Maidstone, Kent, UK) were impregnated with different amounts of extract (10 μL, 20 μL, 30 μL, 40 μL, 60 μL, 80 μL and 100 μL) then they were left to air-dry for 4 h [97]. For each test, two discs were prepared as a control: The first impregnated with carrier solvent, the latter with an ‘extraction blank’ represented by MeOH/CHCl3 used as solvent in extraction, then dried and resuspended in ethanol. 100 μL of each microbial suspension (about 10^8^ CFU mL^−1^) were spread [98] under sterile conditions on a specific agarized medium for each bacterial and fungal tested strain; the Petri dishes inoculated with *Vibrio* species were incubated at 30 °C, those with human pathogenic strains at 37 °C. The antibacterial activity resulted by the evidence of a clear zone around the discs indicating the microbial growth inhibition. Then, the diameter of this clear zone was measured in millimeters. Since from the NMR and GC analyses the α-linolenic acid resulted in the most abundant fatty acid, this pure compound (≥99, Sigma-Aldrich, Oakville, ON, Canada) was employed in ethanol to determine its effective antimicrobial activity. In particular 0.009 mg mL^−1^, 0.018 mg mL^−1^, 0.035 mg mL^−1^, 0.051 mg mL^−1^, 0.067 mg mL^−1^ and 0.082 mg mL^−1^ were tested for antibacterial action.

### 4.5. Antioxidant Activity

#### 4.5.1. Oxygen Radical Absorbance Capacity Assay (ORAC)

For ORAC, the method of Dávalos et al. [99] was used. The assay was performed using 75 mM phosphate buffer (pH 7.4) in black-walled 96-well plates (Greiner-Bio One, Frickenhausen, Germany) and each well contained a final volume of 200 μL. A volume of 20 μL of extract and 120 μL of fluorescein (FL; 70 nM final concentration) were added into the wells and the plate was incubated at 37 °C for 15 min. The AAPH (60 μL; 12 mM final concentration) was added and fluorescence intensity (λ_EX_ = 485, λ_EX_ =535) was estimated using an Infinite200 Pro plate reader (Tecan, Männedorf, Switzerland), every minute for a total of 80 min. A standard curve was constructed using 6-hydroxy-2,5,7,8-tetramethylchroman-2-carboxylic acid (Trolox, Sigma-Aldrich, Oakville, ON, Canada, 1.5–10.5 μM). A blank (fluorescein + AAPH) using phosphate buffer instead of the antioxidant solution was carried out in each assay. Results were determined on the basis of the difference in area under the curve between the control and the sample and expressed as micromoles of Trolox equivalents (TE) per g of lipidic extract. All the reaction mixtures were prepared in triplicate and at least three independent assays were performed for each sample.

#### 4.5.2. Trolox Equivalent Antioxidant Capacity Assay (TEAC)

The TEAC assay was performed adapting the method described by Re et al. [100] to a microplate reader. 2,2′-Azinobis (3-ethylbenzothiazoline-6-sulfonic acid) diammonium salt (ABTS, Sigma-Aldrich, Oakville, ON, Canada) radical cations were prepared by mixing potassium persulfate 2.45 mM (final concentration) and an aqueous solution of ABTS 7 mM (final concentration) in the dark at room temperature for 12–16 h. The ABTS radical cation solution was diluted in PBS (pH 7.4) to an absorbance of 0.40 at 734 nm ± 0.02. A standard calibration curve of Trolox (0–16 μM) was constructed. A volume of 10 μL of Trolox or extracts diluted in PBS were added in the wells of a 96 well-plate (Costar, MERCK, Darmstadt, Germany) with 200 μL of diluted ABTS. Afterwards the absorbance reading at 734 nm was taken 6 min after initial mixing using an Infinite200 Pro plate reader (Tecan, Männedorf, Swizerland). Appropriate solvent blanks were run in each plate. The lipidic extract was assayed in at least three separate dilutions and in triplicate. The inhibition of absorbance at 734 nm of the lipidic extract was plotted as a function of concentration of Trolox and the TEAC value expressed as Trolox equivalent (in micromolar) per g of lipidic extract, using Magellan v 7.2 software (Tecan, Männedorf, Switzerland).

#### 4.5.3. Folin–Ciocalteu (F–C) Assay

A microplate methodology was used to perform a Folin–Ciocalteu assay in algae lipidic extract [101]. The assay was performed in microtriter 96-well plates (96-well clear round bottom plate, Corning) using a microplate reader (Tecan, Infinite M200, Männedorf, Switzerland). Gallic acid standard solution or extract sample (50 μL) and a Folin–Ciocalteu Reagent (FCR; 1:5, *v*/*v*; 50 μL) were placed in each well then, 100 μL of sodium hydroxide solution (0.35 M) was added. The absorbance at 760 nm was monitored after 5 min. To evaluate the absorption of sample, 50 μL of 0.4 M of acid solution was added instead of the FCR. A blank was evaluated by the addition of 50 μL of water instead of standard compound or sample. All experiments were performed in triplicate. A calibration curve of gallic acid in the range from 2.5 mgL^−1^ to 40.0 mgL^−1^ was established (R ≥ 0.9997). The absorbance values obtained for samples were related to those of the gallic acid standard curve and the F–C reducing capacity was expressed as gallic acid equivalents (mgGAE/g lipidic extract).

### 4.6. Gas Chromatographic Analysis of Fatty Acid

The method by Folch et al. [102] was employed in order to extract total lipids from algae. All the samples were homogenized and extracted with methanol/chloroform/water (1/2/1) and a final volume 20 times the sample volume was obtained. Fatty acids composition was established in according to Budge and Parrish [103]. Briefly, the fatty acids (FAs) of total lipids were transesterified to methyl esters accordingly to Stabili et al. [3,4]. The samples were cooled, and then 1 mL of distilled water was added followed by vigorous shaking. Fatty acid methyl esters (FAMEs) were collected in the upper benzene phase. The benzene phase was transferred to a vial and the drying was achieved by a nitrogen stream, with a very slow flow rate, to avoid the loss of the sample. The analyses of sample FAME extracts were performed via gas chromatography using an HP 6890 series GC (Hewlett Packard, Wilmington, DE, USA) equipped with flame ionization detector. FAMEs were separated with an Omegawax 250 capillary column (Supelco, Bellafonte, PA, USA; 30 m long, 0.25 mm internal diameter and 0.25 mm film thickness). The column temperature program was as follows: 150–250 °C at 4 °C/min and then held at 250 °C. FAMEs were identified by comparing retention times obtained with those of known standards (FAME mix, Supelco-USA) and the results were reported as percentages of total identified methyl ester fatty acids. Helium was employed as the carrier gas at a flow of 1 mL/min. The injection volume was 1 mL. All assays were conducted in triplicate

### 4.7. NMR Spectroscopy

The lipid fraction of *C. linum* was characterized by 1D and 2D NMR spectroscopy, with the same methodology already reported in Stabili et al. [3]. 1D ^1^H and 2D ^1^H *J*res, ^1^H–^1^H COSY, ^1^H–^13^C HSQC and ^1^H–^13^C HMBC spectra were recorded at 298 K on a Bruker Avance III NMR spectrometer (Bruker, Milan, Italy), operating at 600.13 MHz for ^1^H observation, equipped with a TCI cryoprobe incorporating a z axis gradient coil and automatic tuning-matching (ATM). The lipid extract was dissolved in 600 μL of CD_3_OD/CDCl_3_ (1:2 mix) and transferred to a 5 mm amber NMR tube, using tetramethylsilane (TMS, δ = 0.00) as an internal standard. The following parameters were used for ^1^H NMR spectrum: 64 K data points, spectral width of 20.0276 Hz, 64 scans with a 2 s repetition delay, 90° power pulse (p1) 7.3 µsec and power level 8.05 dB. The acquisition and processing of spectra were performed using Topspin 3.5 software (Bruker Biospin, Milan, Italy). Resonances of fatty acids and metabolites were assigned on the basis of literature data [3,4,26,31,43,44,45,46,47,48,49,50].

### 4.8. Thin-Layer Chromatography

Data on pigments (chlorophylls and carotenoids) from NMR analysis were also compared with those obtained by thin-layer chromatography (TLC) analysis. Chromatography silica gel TLC-plates were performed on the algal lipidic extracts and developed at room temperature with an eluent solution of hexane and acetone (3:2). Pigments revealed from TLC were identified by retention factors (Rf) and literature data [104,105].

### 4.9. Chaetomorpha Linum Cultivation Trials

In order to obtain high algal biomass availability to extract the evidenced bioactive compounds, *C. linum* cultivation trials were realized in an (IMTA) system with fish cages. About 20 kg of seaweed were collected from the Mar Piccolo and transferred to an aquaculture farm (Maricoltura Margrande, Taranto, Italy) located in a coastal site of the near Mar Grande (northern Ionian Sea). Here, seaweeds were inserted in net sacks, which were hung for cultivation at 1 m of depth within a long-line system, with *Mytilus galloprovincialis*, polychaetes and porifera around fish cages (Figure 5).

## Figures and Tables

**Table 1:** Antimicrobial activity of *Chaetomorpha linum* lipidic extract.

Microbial Strain	Diameter of Growth Inhibition (mm)								
	1 μL	5 μL	10 μL	20 μL	30 μL	40 μL	60 μL	80 μL	100 μL
*Candida albicans*	0	0	0	0	0	0	0	0	0
*Candida famata*	0	0	0	0	0	0	0	0	0
*Candida glabrata*	0	0	0	0	0	0	0	0	0
*Enterococcus* sp.	0	0	0	0	0	0	0	0	0
*Pseudomonas* sp.	0	0	0	0	0	0	0	0	0
*Staphylococcus* sp.	0	0	0	0	0	0	0	0	0
*Streptococcus agalactiae*	0	0	0	0	0	0	0	0	0
*Vibrio alginolyticus*	0	0	0	0	0	0	0	0	0
*Vibrio harveyi*	0	0	0	0	0	0	0	0	0
*Vibrio mediterranei*	0	0	0	0	0	0	0	0	0
*Vibrio ordalii*	0	8	12	12	12	12	12	12	12
*Vibrio parahaemolyticus*	0	0	0	0	0	0	0	0	0
*Vibrio salmonicida*	0	0	0	0	0	0	0	0	0
*Vibrio vulnificus*	0	8	12	12	12	12	12	12	12

**Table 2:** Antioxidant activity of *C. linum* lipidic extract assayed by Trolox equivalent antioxidant capacity (TEAC) and oxygen radical absorbance capacity (ORAC) assays.

TEAC (μmolTE/g Extract)	ORAC (μmolTE/g Extract)	Folin–Ciocalteu (mgGAE/g Extract)
30.554 ± 2.297	170.960 ± 16.830	5.867 ± 0.136

Data are the mean ± SD (*n* = 3).

**Table 3:** Chemical shifts ^1^H (ppm), ^13^C (ppm) and assignments of metabolite resonances in the ^1^H NMR spectrum of algal lipid extract (CHO—cholesterol, FA—fatty acids, SFA—saturated fatty acids, UFA—unsaturated fatty acids, ARA—arachidonic acid, DHA—docosahexaenoic acid, PHB—poly-β-hydroxybutyrate, DUFA—diunsaturated fatty acids, PUFA—polyunsaturated fatty acids, MAGs—monoacylglycerols, DAGs—diacylglycerols, TGs—triacylglycerols).

Compound	Assignment	δ^1^H (ppm, Multiplicity)	δ^13^C (ppm)
CHO	–CH_3_-18	0.68 (s)	11.67
	–CH_3_-26	0.86	22.45–22.38
	–CH_3_-21	0.92 (d)	18.56
	–CH_3_-19	1.01 (s)	19.06
All FA (SFA, UFA)	–CH_3_	0.97–1.02	14.18
All FA	–(CH_2_)– COOCH_2_CH_2_	1.22–1.33 (m) 1.46–1.68 (m)	
UFA	CH_2_=CH_2_–CH_2_	1.98–2.07	27.2
All FA	CH_2_–C=O	2.32–2.38	
ARA	CH_2_–COOH	2.38	
DHA	CH_2_–CH_2_–COOH	2.38–2.42	22.59
			34.07
PHB	CH_3_	1.26	
	CH	5.23	
	CH_2_	2.48 (dd)	40.75
	CH_2_	2.58(dd)	169.9
DUFA	CH_2_	2.73–2.78	25.6
PUFA ω-3 (DHA, linolenic acid)	CH_2_	2.78–2.86	
MAGs	CHOCO	3.63	70.5
DAGs	OH–CH_2_–CH	3.73 (m)	
	2’CHOCO	5.08 (m)	
TGs	CH_2_ (sn1,3)	4.15	62.01
	CH_2_ (sn1,3)	4.28	
	CH (sn2)	5.26	
All UFAs	CH=CH	5.30–5.42 (m)	68.6
Dehydroabietic and abietic acids	CH	6.88	125.57
	CH	7.00	127.28
	CH	7.16	
Alkaloid species		7.53	130.88
		7.72	128.90
Chlorophylls a	CH-20	8.55	
	CH-5	9.54	
Chlorophylls b		9.83	
	CH-5	9.99	
		10.04	
	CHO-7	11.23	
		11.25	
Pheophytin a	CH	9.35	
		9.40	
Pheophytin b	CH	9.60	
		9.62	
Lutein	CH=CH	6.13–6.32	
β-carotene	CH=CH	6.57–6.69	
